# Three-Dimensional Simulation of DRIE Process Based on the Narrow Band Level Set and Monte Carlo Method

**DOI:** 10.3390/mi9020074

**Published:** 2018-02-09

**Authors:** Jia-Cheng Yu, Zai-Fa Zhou, Jia-Le Su, Chang-Feng Xia, Xin-Wei Zhang, Zong-Ze Wu, Qing-An Huang

**Affiliations:** 1 Key Laboratory of MEMS of the Ministry of Education, Southeast University, Nanjing 210096, China; 220151251@seu.edu.cn (J.-C.Y.); 220161278@seu.edu.cn (Z.-Z.W.); hqa@seu.edu.cn (Q.-A.H.); 2 Central Semiconductor Manufacturing Corporation, Wuxi 214061, China; sujl@csmc.crmicro.com (J.-L.S.); xiacf@csmc.crmicro.com (C.-F.X.); zhangxw@csmc.crmicro.com (X.-W.Z.)

**Keywords:** deep reactive ion etching, level set method, Monte Carlo simulation, ray tracing algorithm, surface evolution

## Abstract

A three-dimensional topography simulation of deep reactive ion etching (DRIE) is developed based on the narrow band level set method for surface evolution and Monte Carlo method for flux distribution. The advanced level set method is implemented to simulate the time-related movements of etched surface. In the meanwhile, accelerated by ray tracing algorithm, the Monte Carlo method incorporates all dominant physical and chemical mechanisms such as ion-enhanced etching, ballistic transport, ion scattering, and sidewall passivation. The modified models of charged particles and neutral particles are epitomized to determine the contributions of etching rate. The effects such as scalloping effect and lag effect are investigated in simulations and experiments. Besides, the quantitative analyses are conducted to measure the simulation error. Finally, this simulator will be served as an accurate prediction tool for some MEMS fabrications.

## 1. Introduction

For the fabrication of high aspect ratio MEMS structures, chemical etching, and plasma sputtering are not chosen due to the isotropic characterization and bad selectivity. As the only recognized production deep reactive ion etching (DRIE) process, the Bosch process is introduced to enable a high aspect ratio by plasma etching and passivation alternation in multiple cycles [1,2]. The etchants like SF_6_ and NF_3_ generate the free radicals of fluorine which will react with the silicon substrate. Then, a thin layer of fluorocarbon polymer is deposited to protect the lateral sidewalls.

On account of the complicated behaviors of particles such as sticking, scattering, diffusion, recombination, and redeposition in DRIE process, a synthetic and topographical model is adopted to sketch the complex mechanism. For example, the simulation surface profiles are described by narrow band level set method [3,4]. The Monte Carlo method depicts the physical mechanism such as sticking and re-emission. The simulation domain contains 300 × 300 × 300 grids. The balance between runtime and accuracy is fully considered.

Since DRIE is the key procedure to fabricate the structures with high aspect ratio, several simulators have already been developed. A two-dimensional simulator was developed by Zhou et al. using string-cell hybrid method to track the evolving surface [5]. However, the extension to 3-D is difficult. Later Li et al. presented a simplified geometric model which used visible angles to calculate the flux [6]. This simulator is fast, but not so accurate. Based on Li’s framework, the advanced simulator which adds a Monte Carlo particle simulation has three advantages. On the one hand, the simulation results are not identical compared with the geometric level set model, which corresponds to the subtleties in the manufacture. On the other hand, the selective etching ratio, such as the substrate-mask etching ratio, can be simulated by extracting the parameters in different materials. Finally, it can also simulate the Silicon-on-Insulator (SOI) DRIE process, which can be applied to fabricate the comb actuators, accelerometer, and piezoelectric structures. Table 1 shows the characteristics of different simulators.

## 2. Models of Three-Dimensional DRIE Process

### 2.1. Theoretical Model of DRIE Process

**Figure 1:**
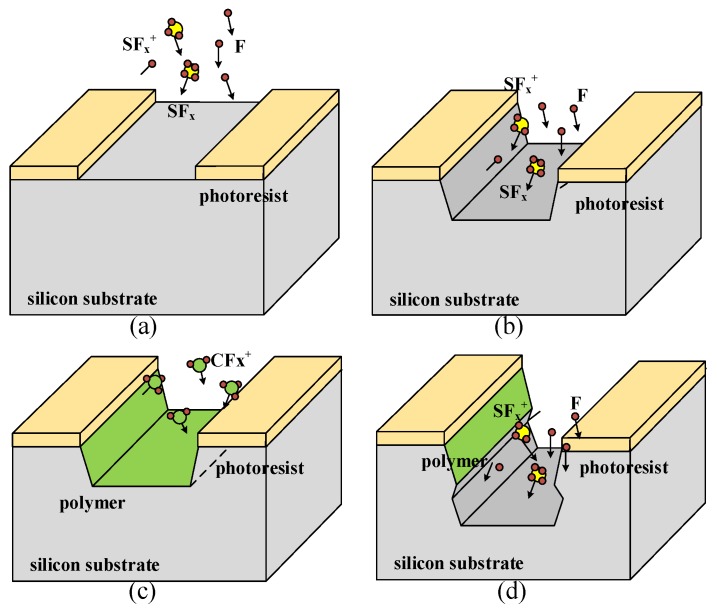
Schematics of the principle of the DRIE process. (**a**) The initial state. (**b**) The first etching process. (**c**) The upcoming passivation. (**d**) The second etching process.

As mentioned above, DRIE process is composed of multiple cycles of etching/passivation process. The basic principles of DRIE are shown in the Figure 1. In the radio frequency (RF) glow discharge, sulfur hexafluoride (SF_6_) is dissociated and ionized into massive species. The dominant neutral particles (SF_6_, SF_4_, F_2_, F), positive ions (${\mathrm{SF}}_{5}^{+}$, ${\mathrm{SF}}_{3}^{+}$) and negative ions (F^−^) are determined in the previous work [7,8]. While in our simulator, there are neutral and ionized type of particles with different angular and velocity distribution. The differences of particles in the same type are neglected because it is unclear to obtain the contribution of etching rate.

**Figure 2:**
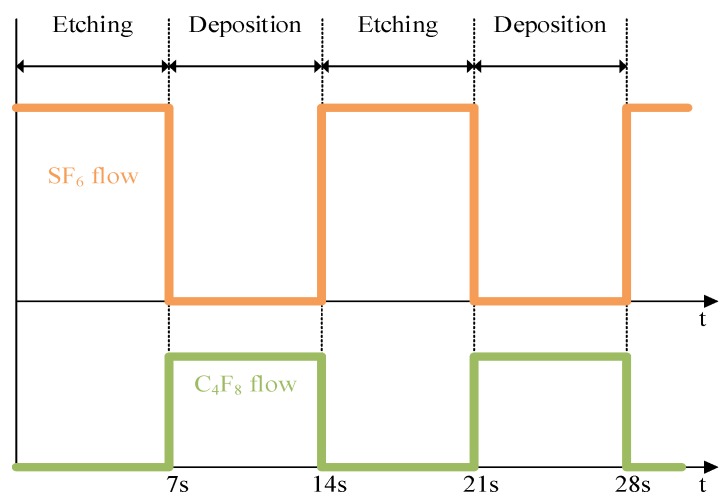
Schematic of alternating etching/passivation flow.

The silicon substrate is isotropically etched by these active particles shown in Figure 1b. Ar gas stabilizes the glow discharge in ICP-RIE system. Ten percent O_2_ as an assistant gas prohibits the combinations of active radical sites SF_x_ and F in plasma chamber. Additionally, O_2_ can also provide oxygen in the passivation layer. At the same time, physical sputtering of neutral particles also exists [9]. Then, during the passivation step, a type of fluorocarbon gas like C_4_F_8_ is used to grow a thin protection film of nearly 10 nm. This fluoride polymer impedes the reaction between F radical sites and Si substrate shown in Figure 1c. During the next etching cycle, the lateral polymer persists due to the low impact probability of directional ions while the bottom polymer is removed. By alternating the etching and passivation period in Figure 2, the direction of etching will only occur along the vertical side shown in Figure 1d.

### 2.2. The Main Steps of Simulation

**Figure 3:**
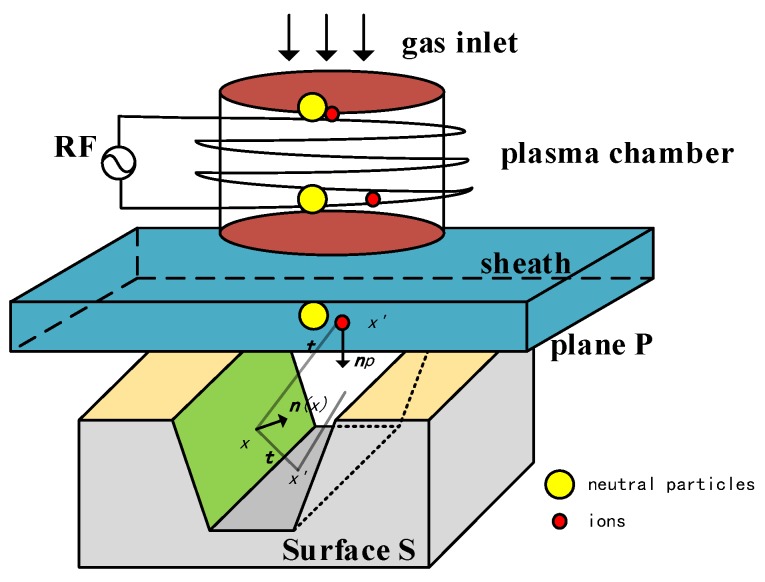
Schematic of three main phases in PECVD or plasma enhanced etching.

The total process of DRIE process incorporates three main parts shown in Figure 3. The first phase is to simulate the generation of plasma including the physical phenomena like dissociation, movements in potentials and molecular collision. The distributions of velocity, energy, and direction of different particles should be obtained from stage one. The second phase is the ballistic transport from the bottom of sheath plane P to surface S. The etching/passivation rate is assumedly determined in phases one and two. Phase three is to solve the level set equation according to the etching/passivation rate mapping. Finally, with the information stored in material matrix of every grid, and the profiles including photoresist pattern are illustrated in the simulator.

Due to the complexity of building the model of the plasma sheath, an empirical formula is introduced to describe the quasi-static plane P. Equation (1) contains the flux distribution, incident angles and energy distribution [10]:

(1) $$\Gamma^{source}\left(t,E\right)=\Gamma^{source}\frac{\alpha +1}{2\pi }{\left(t\cdot n_{p}\right)}^{\alpha }f\left(E\right)$$

where the neutral and ionic reactants are introduced in source plane P. The angle distribution is distinguished by parameter *α* in Equation (1). In terms of ions, *α* is about 100, which presents the Gaussian-like distribution. The neutral particles correspond to cosine-like distribution since *α* = 1 [11]. Additional, here $\Gamma^{source}$ is total fluxes of ions and neutrals, t denotes incident direction and $n_{p}$ is the vertical vector pointing to surface S, *f*(*E*) is the distribution function of energy.

After generating plenty of particles in the random model, the coordinates and directional vectors are stored in the memory. All of these particles should be traced until they interact with surface S. The Monte Carlo probabilistic approach is used to describing the trajectory of all particles, which generates a relatively large sample to predict the system. Due to the low pressure in the system, the mean free path will be larger than the feature dimension of system. The collision between two particles can be neglected. In addition, the surface charging effect is also negligible. Thus, the flux function on the surface S is connected with the flux in the plane P by Equation (2):

(2) $$\Gamma^{surface}\left(x,t,E\right)=\frac{-t\cdot n\left(x\right)}{{\left|x-x^{\prime }\right|}^{2}}\Gamma^{source}+\frac{-t\cdot n\left(x\right)}{{\left|x-x^{\prime }\right|}^{2}}\Gamma^{reflection}$$

where vector ***t***, ***n***(***x***) are incident direction and normal vector of the surface. |***x*** − ***x*′**|^2^ is the distance. Considering the re-emission, the surface flux includes the flux from source plane P and reflective flux with sticking factor *η*. The velocity function Equation (3) on the surface is derived by the surface flux and yield function *Y*:

(3) $$F\left(x\right)=\int \Gamma^{surface}\cdot Y\left(n\left(x\right);t\right)d\Omega$$

## 3. Simulation Methods

### 3.1. Narrow Band Level Set Method

Firstly, in order to describe the shapes of fronts in each time step, multiple profile evolution algorithms are developed. In etching and deposition process, string algorithm, cell-based method, and the level set method are used frequently [12,13]. Multiples advantages and disadvantages are weighed in the publications before. In this paper, the level set techniques and its advanced version inspired by Sethian will be applied to this simulator [14,15]. Level set method can provide gradients of local surface, signed distance function and Courant–Friedrichs–Lewy (CFL) condition which avoids the instability. Moreover, level set technique facilitates the topographical changes with less memory and computation.

**Figure 4:**
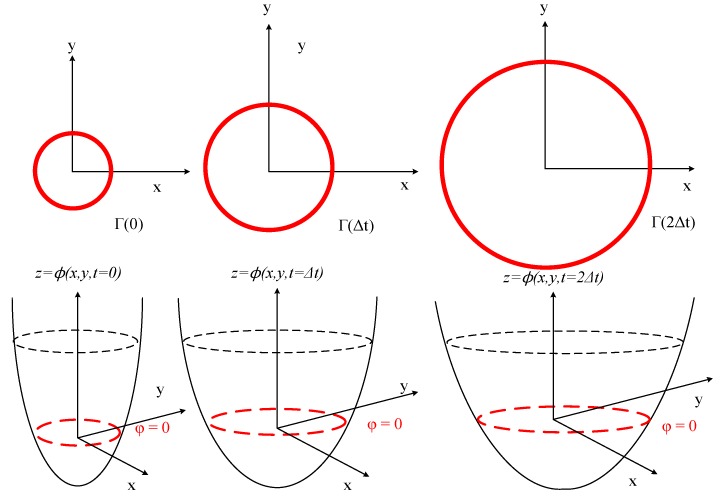
Illustrations of 2-D propagation of Γ(t) with the moving level set function φ.

Considering a moving hypersurface Γ(t), embedded in a one-dimensional higher time dependent level set function φ(x, t). And the boundaries are always determined as the zero level set φ(x, t=0) in Equation (4) shown in the Figure 4:

(4) Γ(t)={x|φ(x, t)=0}

The evolving level set function Equation (5) is deduced by chain rules with the initial signed distance function Equation (6):

φ(x(t), t)=0

$$\varphi_{t}+\nabla \varphi \cdot x^{\prime }\left(t\right)=0\;\mathrm{with}\;x_{t}\cdot \nabla \varphi =F\left(k\right)\cdot \left|\nabla \varphi \right|$$

(5) $$\varphi_{t}+F\cdot \left|\nabla \varphi \right|=0$$

(6) φ(x,t=0)=±d(x)

We can solve Equation (6) of each grid nodes by differential scheme Equation (7) with “entropy-satisfying” approximation to the gradient Equation (8). The zero level set surface can be extracted within φ<0.5:

(7) $$\varphi_{ijk}^{n+1}=\varphi_{ijk}^{n}-\left[max\left(F_{ijk},0\right)\nabla^{+}+min\left(F_{ijk},0\right)\nabla^{-}\right]\cdot h$$

(8) $$\nabla^{+}={\left[max{\left(D_{ijk}^{-x},\;0\right)}^{2}+min{\left(D_{ijk}^{+x},\;0\right)}^{2}+max{\left(D_{ijk}^{-y},\;0\right)}^{2}+min{\left(D_{ijk}^{+y},\;0\right)}^{2}+max{\left(D_{ijk}^{-z},\;0\right)}^{2}+min{\left(D_{ijk}^{+z},\;0\right)}^{2}\right]}^{\frac{1}{2}}\;\nabla^{-}={\left[max{\left(D_{ijk}^{+x},\;0\right)}^{2}+min{\left(D_{ijk}^{-x},\;0\right)}^{2}+max{\left(D_{ijk}^{+y},\;0\right)}^{2}+min{\left(D_{ijk}^{-y},\;0\right)}^{2}+max{\left(D_{ijk}^{+z},\;0\right)}^{2}+min{\left(D_{ijk}^{-z},\;0\right)}^{2}\right]}^{\frac{1}{2}}$$

**Figure 5:**
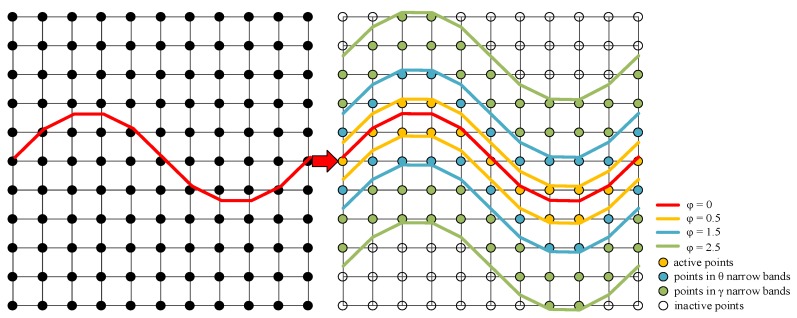
Illustrations of 2-D simulation domain for all spacing grids and the three-layer narrow band.

Originally the level set method should store φ all grid spacing. However, this simulator uses the narrow band level set method to decrease storage requirements [3,16] shown in the Figure 5. Since the level set values of points far away zero level set will not be considered to upgrade or reinitialize. Namely, at every time step the level set values of points inside the narrow band will be upgraded according to the surface velocity. The procedure called re-initialization is operated after upgrading all values in narrow band. Additionally, the narrow band is reconstructed according to the latest level set values. However, this simplification should guarantee that the propulsion of surface avoids exceeding the *γ* narrow bands.

The computation complexity of this straightforward level set method is O(N^3^). N is the number of grid points in one dimension. While the narrow band method reduces to O(kN^2^) where k is the width of narrow band. The three-layer narrow band confines the boundaries well, which alerts whether the narrow band should be reconstructed. A significant reduction of memory usage and calculation has been seen as fewer grids participate in the calculation.

### 3.2. Monte Carlo Method Accelerated by Ray Tracing

From Equations (6) and (8), the velocity $F_{ijk}$ should be determined. Sethian et al. utilize the velocity function to simulate the isotropic and anisotropic etching (deposition) such as F(θ)=1, F(θ)=cosθ or $F\left(\theta \right)=\cos^{10}\theta \cdot \sin\theta$ [17]. Additionally, Li et al. find the local flux by geometric visibility. However, in this simulator, the physical particle transport and surface kinetics are involved to calculate the local flux. Based on the local flux function Equation (3) and velocity integral Equation (4), the direct solution is to discretize the surface integral, which leads to a dense system matrix.

Hence, a ray tracing algorithm is applied to realize the realistic picture for one particle [18,19,20]. Additionally, the Monte Carlo statistical method allows millions of rays to represent the behaviors of up to 10^18^ particles in the real situation. Therefore, the second phase is simplified by a ray tracing technique, like rendering in in three dimensions. Millions of particles per second are computed analogously to determine the contributions of velocities in each grid when these particles hit the surface. To enable the calculation of local flux, the first intersections between rays and narrow band surface are found. Since the source plane P and level set surface are meshed into grids, it is difficult to detect the intersections between rays and grids. The bounding box is then presented. As sphere is the most popular and least complicated primitive bounding volume, the level set surface is approximated with multiple spherical surface in this simulator. As we know, the information of active grids like level set function φ, coordinates and material matrix are stored in computer memory. The real surface is described by these active grids. Thus, the average distances between the grids and the real surface should be the radius of sphere. The radius is set as the half grid length *r* = 0.5 accordingly. The local flux is mapped to intersection impacts on millions of particles.

**Figure 6:**
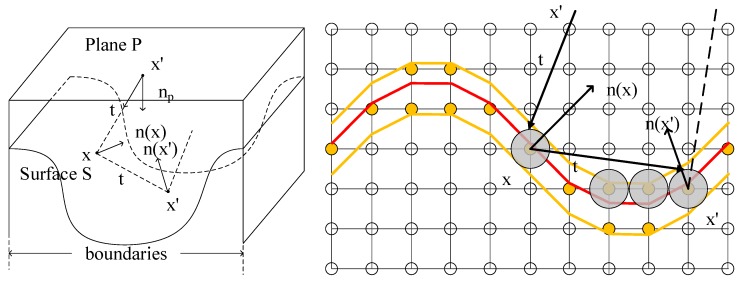
Illustrations of particle trajectory intersecting with group of active spheres.

With the spheres bounding the active points, the first intersections are determined in Figure 6. The basic steps of ray tracing algorithm are presented [20]:

- Step 1: Calculate the distance d1 between origin ***x*′** and center ***x***.
- Step 2: Calculate the closest distance d2 between ray and center ***x***.
- Step 3: Find square of half chord intersection distance d3.
- Step 4: Test if square is negative (no intersection).
- Step 5: Compare the distance d1 and choose the minimum (first intersection).
- Step 6: Find the normal vector.
- Step 7: Calculate the reflective direction.

The bridge between the level set method and the Monte Carlo method is the local velocity calculation. As the Monte Carlo method is utilized, the integral Equation (4) is discretized automatically. In order to save the calculation time without losing accuracy, the etching part is decomposed to isotropic rate mainly caused by neutral particles and anisotropic rate assisted by ion particles. The etching rate is depicted as a linear combination of neutral and ion flux. Therein the parameters *C_uni_* and *C_ion_* in Equation (9) are dependent on different materials exposed in the etching gas. However, the etching rate Equation (9) is invalid when there are only ions or neutrals. The next passivation rate can be simplified as a constant *C_d_*. Thus, the rates of etching and deposition in DRIE process can be denoted as *ER* and *DR* in Equations (9) and (10):

(9) $$ER=C_{uni}\Gamma_{n}+C_{ion}\Gamma_{i}$$

(10) $$DR=\;C_{d}$$

The *ER* and *DR* are solved by Equations (9) and (10) and substituted in a differential scheme Equation (7). Thus, the coupling LS-MC method of DRIE is completed.

## 4. Simulation and Experimental Results

**Figure 7:**
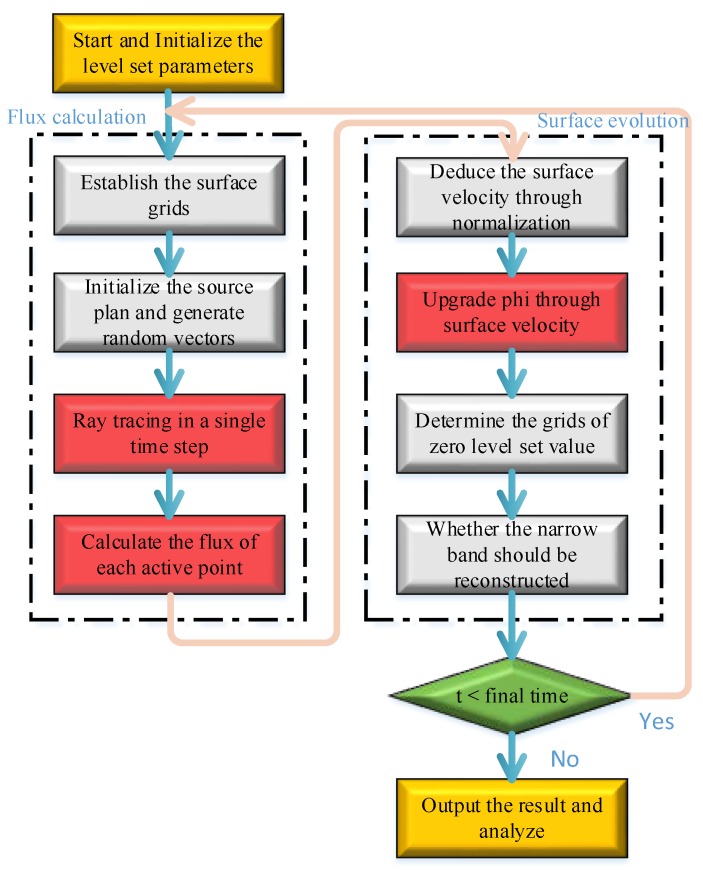
The flowchart of coupling LS-MC method.

The flowchart which couples LS-MC are demonstrated in the Figure 7. The time step is not a constant restrained by CFL condition Equation (11). In other words, the time steps are dependent on the maximum velocity in all active grids. And in each step, the maximum velocity is varied owing to the randomicity of Monte Carlo:

(11) $$\frac{\Delta t}{max\left\{\Delta x\right\}}max\left\{F\right\}\leq 1$$

The initial geometry is built by sign distance function of φ and the material matrix. Given the initial time step and the number of particles per second in the simulation domain, a loop of flux calculation and surface evolution is started after reading the layout. Until the time is greater than the final etching time, one cycle of etching is stopped. The number of cycles can be set to obtain the repetitive simulation. After finishing all cycles, the marching cube algorithm [20,21] is applied to extract the material matrix. The matrices of air, silicon, mask, passivation polymer, and insulator in SOI are read to visualize the profiles of interface level set.

### 4.1. Qualitative Analysis

**Figure 8:**
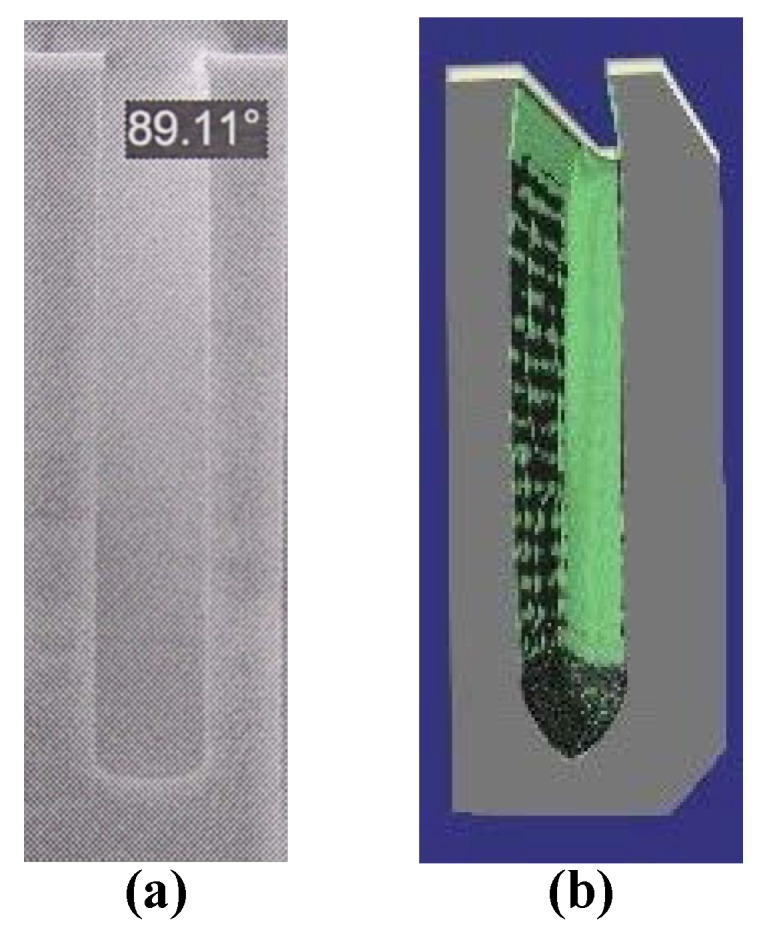
The SEM photography (**a**), and the simulation result (**b**) of a single trench.

A simulation of Bosch process etching a 5 μm wide trench has been conducted. The alternating cycle of etching and passivation is 7s/7s. Figure 8a shows the deep vertical holes in SEM photography while Figure 8b illustrates the simulation results. The scalloping effects are clearly seen in the simulation Figure 8b. In practice, to reduce the roughness (nearly 100 nm), the sample is processed in the potassium hydroxide (KOH) and isopropyl alcohol (IPA) by chemical wet polishing etching. Thus, the sidewall of SEM diagram is relatively smooth. In the simulation domain 300 × 300 × 300 grids, each grid represents 0.1 μm. The thickness of the mask is 0.5 μm. Finally, the typical CPU runtime of 20 cycles is nearly 20 h on Intel (R) E5-2630 @2.2GHz (Gentai, Shanghai, China) and the usage of memory is about 560 MB. Since the material and level set values for each grid should be stored.

**Figure 9:**
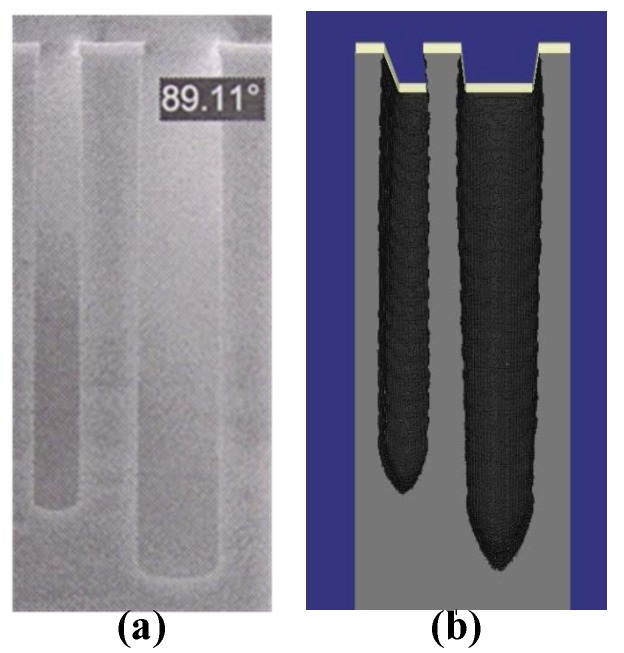
The SEM photography (**a**) and simulation result (**b**) showing lag effect.

Furthermore, the loading effect should be considered in this simulator [22]. Usually the aspect ratio dependent etching or RIE lag influence the depth of the trench due to its own sizes exposed to the plasma. To put it another way, the larger holes will have deeper length in the same ambience. Figure 9 illustrates the SEM photography and simulation profiles simultaneously. The simulation domain is still resolved on the 300 × 300 × 300 grids. Compared with the SEM picture of two trenches (2.5 μm and 5 μm), the lag effect is clearly seen. And the etching rate decreases with the increment of aspect ratio. Since there is no charge accumulation at the side bottom in simulation model, we can see the different bottom profiles compared to the experiments.

### 4.2. Quantitative Analysis

**Figure 10:**
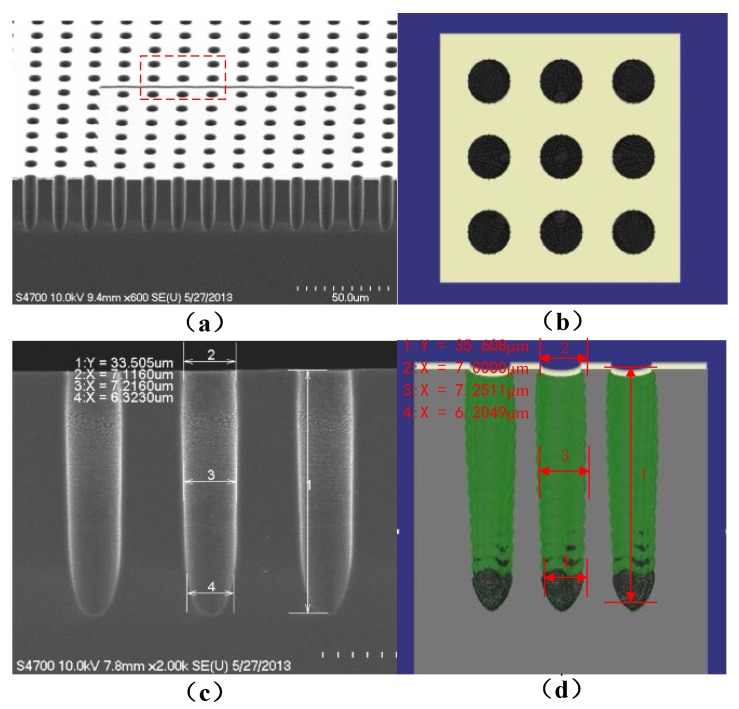
The experimental photography (**a**,**c**) and simulation (**b**,**d**) of porous structure.

After extracting the parameters from the fabrication, the number of particles of each grid is 80, so the total number of particles in source plane P is 0.8 million per second in the same domain (300 × 300 × 300) for Monte Carlo method. Figure 10 shows that the mask of replicated domain is chosen to reduce the calculation without simulating the whole die. Table 2 shows the experimental and simulation parameters and the errors are computed.

**Figure 11:**
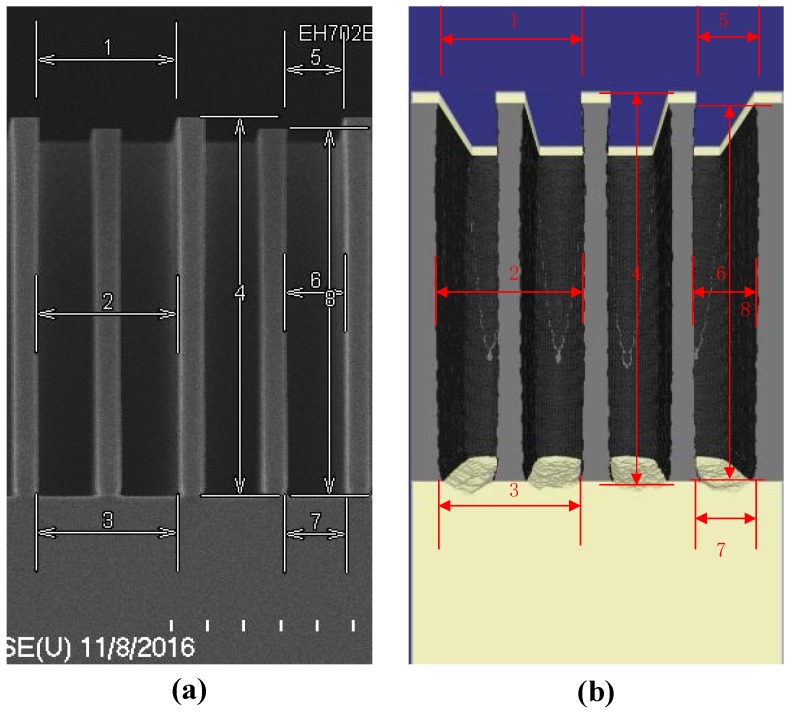
The experimental SEM photography (**a**) and simulation (**b**) of deep trenches in SOI structure.

The second experiment is to etch the deep trenches on a specified wafer [23,24]. SOI wafer (silicon on insulator) is composed of two silicon layers with one silicon dioxide layer inside. The top layer called device layer will be etched to establish the complex structures. Due to the isolation of silicon dioxide, the reactive ion etching stops at the interface. The experimental and simulation results are shown in the Figure 11. From the 3-D simulation result Figure 11b, the interface is rough because of the particles sputtering. And the typical sizes of trenches and aspect ratio are given in Table 3. The average error of simulation compared with the experiments is 2.419%.

### 4.3. Accuracy and Runtime

**Figure 12:**
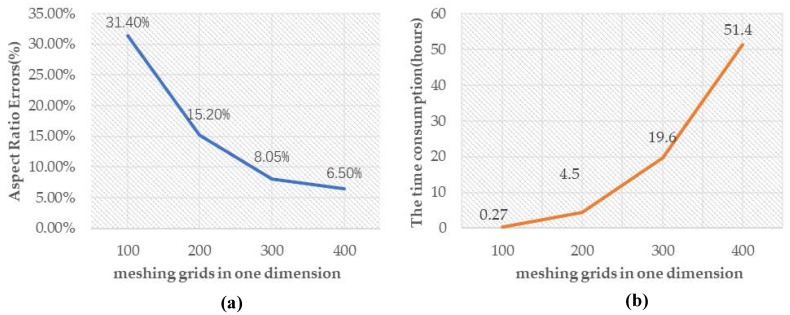
Line charts of aspect ratio errors (**a**) and runtime (**b**) simulating the porous structures in different resolutions.

The simulation accuracy and runtime are strongly dependent on meshing. In this simulator, we can implement the same simulation with different resolution. Usually the grids range from 50 to 500 in each direction. Based on the layout of porous structure in Figure 10, the dimension is 50 μm × 50 μm × 50 μm. The 100, 200, 300, and 400 grids are chosen to simulate at the same physical parameters. Figure 12 shows that the aspect ratio errors and time consumption changes in different resolution. As a result, we choose 300 × 300 × 300 as the final simulation domain with high-precision and acceptable runtime.

## 5. Conclusions

This paper presents a coupling LS-MC model for the DRIE process. The agreement between the simulation results and experimental profiles verifies the correctness of the coupling algorithm. Within the proper simulation domain, the error is less than 15%. At the expense of accuracy, the computation complexity will dramatically drop by reducing the grids. Thus, Monte Carlo method is extremely useful to detect the variations of physical parameters. The developed simulator can serve as an accurate prediction tool for some MEMS fabrications.

## Figures and Tables

**Table 1:** Comparisons among the different simulators for the Bosch process.

	R. Zhou	X. Li	O. Ertl and S. Selberherr	This Simulator
Dimension	2D	3D	3D	3D
Meshing	-	100 × 100	500 × 140	300 × 300
Methods	String-cell hybrid	Narrow band level set	Sparse field level set and ray tracing with disks	Narrow band level set and ray tracing with spheres
Runtime	Not Presented	Half a hour	About two days	19.6 h

**Table 2:** Typical results of deep holes in different locations.

Locations and Items	Sizes in Experiments	Sizes in Simulation	Errors (%) |(Ee−ES)Ee|
Location 1	33.505 μm	35.608 μm	6.277%
Location 2	7.1160 μm	7.0000 μm	1.630%
Location 3	7.2160 μm	7.2511 μm	0.4864%
Location 4	6.3230 μm	6.2049 μm	1.868%
Aspect Ratio	4.7084	5.0869	8.050%

**Table 3:** Typical results of deeps trenches in different locations.

Locations and Items	Sizes in Experiments	Sizes in Simulation	Errors (%) |(Ee−ES)Ee|
Location 1	19.114 μm	18.504 μm	3.191%
Location 2	19.132 μm	19.200 μm	0.355%
Location 3	19.132 μm	18.527 μm	3.162%
Location 4	52.844 μm	52.026 μm	1.548%
Location 5	8.056 μm	8.000 μm	0.695%
Location 6	8.254 μm	8.287 μm	0.400%
Location 7	8.452 μm	7.926 μm	6.223%
Location 8	51.495 μm	49.548 μm	3.781%
Aspect Ratio	6.392	6.194	3.098%
